# Exploring the pregnant women’s perspective of late booking of antenatal care services at Mbekweni Health Centre in Eastern Cape, South Africa

**DOI:** 10.4102/phcfm.v10i1.1300

**Published:** 2018-07-02

**Authors:** Ramprakash Kaswa, George F.D. Rupesinghe, Benjamin Longo-Mbenza

**Affiliations:** 1 Department of Family Medicine, Walter Sisulu University, South Africa; 2 Department of Research, Walter Sisulu University, South Africa

## Abstract

**Background:**

Antenatal care (ANC) services are the gateway for integrated management of several conditions that adversely affect the mother and foetus. More stillbirths than neonatal deaths in South Africa are a reflection of poor quality ANC services.

**Aim:**

The primary aim of this study was to explore the reasons for late booking, and also to determine pregnant women’s knowledge, perceptions and attitude towards antenatal care services they receive in Mthatha area in Eastern Cape, South Africa.

**Setting:**

This was a qualitative study, conducted at Mbekweni Health Centre in the King Sabata Dalindyebo (KSD) subdistrict municipality of the Eastern Cape Province

**Methods:**

This qualitative study consisted of selected pregnant women who presented after 19 weeks of gestation at Mbekweni Health Centre. Data were collected through two different methods, namely, semi-structured interviews and focus group discussions were used until saturation of the themes were reached. The interviews were transcribed verbatim and thematic analyses were undertaken.

**Results:**

Twenty women participated in the study. They were diverse in terms of age 18–41 years, gravidity 1–6 and time of ANC booking 20–28 weeks. The interviews identified a variety of personal, service and organisational reasons for late ANC booking. The themes identified for late ANC bookings were: health care system related issues, socio-economic factors, women’s perceptions and knowledge, and failure of family planning services.

**Conclusions:**

Women’s beliefs, knowledge and perceptions regarding antenatal services outweigh the perceived benefit of early ANC visit. The majority of women had lack of knowledge of contraception, early signs of pregnancy, purpose, timing and benefits of ANC visit.

## Introduction

The antenatal care (ANC) provision or regular check-ups during pregnancy through the public health services in modern obstetrics was started during the late 1930s in the United Kingdom and Northern Ireland.^1^ Soon after, a formal labour and delivery care policy was introduced as an integral part of maternity care.^2^ This led to a substantial decrease in the maternal mortality from the mid–part of the 20th century.^1^ International awareness regarding ANC services during pregnancy has grown during the second half of the 20th century.^2,3^

The universal recommendation for first ANC booking is in the first trimester. According to World Health Organization (WHO) systemic review of 2001, the first trimester is the best time for a mother to book her pregnancy.^4^ In 2004 WHO proposed comprehensive integrated ANC for all pregnant women.^5^ ANC is a gateway for integrated management of several conditions that adversely affect the mother and unborn foetus.^5^ ANC consultation also provides an opportunity to prepare mothers nutritional demand during pregnancy, rest, discomforts of pregnancy, safer sexual practices, family planning, feeding advice and new born care.^6,7^

Provisions of free services for pregnant women in South Africa were introduced in the immediate post-apartheid era to improve maternal and perinatal outcomes. The South African National Department of Health (SANDOH) recommended that women should visit ANC clinic as soon as they realise that they are pregnant and definitely before 20 weeks of gestation. The ANC services should start with women’s first visit irrespective of gestational age. The South African Basic Antenatal Care guidelines recommend that pregnant women should start their ANC visit at 12 weeks of gestation. Despite the provision of free ANC services, South Africa is still performing poorly compared to other middle-income countries.^8^

In 2010, the SANDOH introduced the ‘re-engineering of primary health care’ initiative, aiming to start community-based health care. The programme is focused on ward-based primary health care outreach services. The efforts of this programme are still not evident where maternal mortality rate is concerned.

Antenatal care is widely accepted as contributing towards improved pregnancy outcomes, with late booking linked to increase foetal, maternal and infants’ morbidity and mortality. Compared to other middle-income countries, South Africa has 97% of ANC coverage.^1^ However, late attendance is still hampering the optimum benefits of ANC services. Sixth report on the confidential inquiries into maternal deaths highlights late booking as one of the patient-related avoidable factors.^9^ Because late bookings for ANC have been specifically identified as a cause for concern, this study aimed to understand why pregnant women book late for ANC services at Mbekweni Health Centre in Eastern Cape, South Africa. This study highlighted the importance of context-specific social and cultural factors that are responsible for the delay in initiation of ANC despite free provision of services.

## Methods

### Study design and setting

This was a qualitative study, conducted at Mbekweni Health Centre in the King Sabata Dalindyebo (KSD) subdistrict municipality of the Eastern Cape province. The main language spoken is isiXhosa. Only 4.08% of the populations have medical insurance, meaning that the majority use public health facilities.^10^

### Study population and sampling

The study population consisted of all pregnant women who were attending their first ANC visit after 19 weeks of gestation at Mbekweni Health Centre. During 2015, there were 487 women who attended the ANC services at this facility. The OR Tambo district profile report indicated that 80% of women visited the ANC clinic after 20 weeks of gestation.^10^ The sample was chosen by means of purposive sampling. Pregnant women, 18 years and above were included in study. Women who need urgent medical attention and presented in labour were not eligible for study. The potential participants were identified by an antenatal clinic midwife. Eligible women were given information and asked for voluntary written consent for research participation. We aimed to recruit a variety of participants and to continue the interviews until no new themes emerged.

### The data gathering instrument

We used semi-structured interviews and focus group discussions to gather information. The interview guide was developed from a review of the literature and discussion within the multiprofessional group comprising a family physician, midwives and a general practitioner. A pilot study pretested the key questions to be used during participant interviews as well as the interviewing technique. A 24 year old multigravid participant was interviewed and we transcribed and analysed the data inductively to determine if the research question elicited the desired information. Modifications were made as suggested. The interview guide is summarised in Table 1.

**TABLE 1:** An overview of the semi-structured interview guide.

Theme	Primary question asked
Knowledge of antenatal care booking	What is your understanding of antenatal care booking? When ideally would you like to see a health care worker and why?
Discovery of pregnancy	Tell us about your experience of finding out you were pregnant?
General support during pregnancy	Who you disclosed to first about your pregnancy? What general support you have during your pregnancy?
Acceptance of pregnancy	When you came for your first antenatal care visit? Are there any reasons why you have visited later than usual for antenatal care booking?
Health care services experience	Tell us about your experience of visiting antenatal care clinic. What advice and support you received from clinic?

### Procedure

All interviews were audio recorded and conducted between 01 March 2015 and 31 May 2015 at Mbekweni Health Centre. A Xhosa-speaking trained female research assistant was employed to conduct the semi-structured in-depth interviews. The purpose of the study was explained to the participants in IsiXhosa in order to maximise the respondent rate and to generate reliable information. After obtaining voluntary informed written consent, open-ended questions were asked to guide the participants and additional areas of discussion generated depending on the participant’s responses. All interviewed women were invited on another ANC visit for focus group discussion to achieve broader range of information and clarification. All efforts were taken to conduct the interviews privately and were uninterrupted to ensure that participants spoke freely. All interviews and focus group discussions were recorded on audio tape. No personal identity information was recorded to ensure anonymity.

### Data analysis

We used iterative process of simultaneous data collection, analysis and constant comparison. Research notes and transcripts from audio tapes were transcribed verbatim by a research assistant to facilitate content analysis. A professional nurse from ANC independently coded and analysed the transcribed interviews for accuracy. A systemic framework approach of data analysis, outlined by Mays and Pope, was adopted to identify the themes and categories from the transcribed interviews.^11,12^ All the collected data were arranged in a working order. This involved reviewing and selecting information from field notes and transcripts. The key ideas were selected in order to make a list from the raw data by listening to the audio tapes and reading the research notes. The index data for subsequent retrieval and exploration emerged. At this stage, we revisited the data many times to cross-check or verify the findings and ensure that the conclusions that had been drawn were credible, substantiated by the data and are able to stand up to alternative explanations. The identified themes and categories were then used to support the conclusions of the study.

## Ethical considerations

Written informed consent was obtained from all the participants after informing them about the project in a culturally and linguistically sensitive manner. The confidentiality and anonymity of the participants were assured, and no personal information was disclosed. Ethical revision and clearance was granted by the Walter Sisulu University Human Research and Bioethics Committee (No. 088/2014). Permission was also obtained from the Eastern Cape Department of Health (Ref. EC-2015RP21-973).

## Results

There were 20 pregnant women who participated in this study. All spoke IsiXhosa and were of African ethnicity. The mean age of participants was 28.56 years (18–41). The ANC records were used for gestational age. The mean gestational age at ANC booking was 22.37 weeks (20–28). The mean gestational age at diagnosis of pregnancy was 7.91 weeks (4–16).

Six women were primigravidae, and the parity of multigravidae ranged from two to six. Six were married. All but three had gone up to high school level. Seventeen were unemployed and depended on family and partner for financial support. Six were HIV-positive and two of them were on antiretroviral therapy (ART) even before pregnancy. Six had consulted a traditional healer before booking at ANC clinic and had used over-the-counter traditional remedy during the pregnancy.

Participants were asked for the ideal time for first ANC visit. Fourteen acknowledged that women should book their pregnancy in the first trimester, three in second trimester, one in third trimester and two were not sure. The time lapse from diagnosis of pregnancy to booking at ANC clinic varied from woman to woman. Eleven acknowledged that their first visit to ANC clinic was too late and remaining nine participants were not sure.

### Qualitative data analysis

The key themes were categorised on the basis of pregnant women’s views, experience and issues raised regarding the ANC visits. The reasons for late booking were described under five major themes and these were further categorised into subthemes (Table 1).

**TABLE 2:** Characteristics of pregnant women participants (*n* = 20).

Mean age 28.56 years	
Characteristics	*n*
**Age (years)**	
< 20	4
20–30	8
30–40	7
> 40	1
**Parity**	
Primigravidae	6
Multigravidae	14
**Marital status**	
Married	6
Unmarried	14
**Education level**	
High school and above	17
Primary school	3
**Employment status**	
Employed	3
Unemployed	17
**HIV status**	
Positive	6
Negative	14
**Traditional medicine**	
Yes	6
No	14
**Contraception**	
Yes	6
No	14

*n*, number; mean, average.

**TABLE 3:** Identified themes and subthemes.

Themes	Subthemes
Health system factors	Health care workers’ attitude Long queues at ANC clinics Communication to attend ANC
Patient-related factors	Unplanned and non-acceptance of pregnancy Avoiding HIV testing Fear of parents’ reaction
Socio-economic factors	Transport cost Partner neglects and loss or lack of support Working in another province
Individual perception and knowledge	Experience of previous pregnancy Late booking had less ANC visits No medical problem Other commitments over ANC
Failure of family planning	Using injectable contraception Termination of pregnancy inaccessible

ANC, antenatal care; HIV, human immunodeficiency virus.

### Health system factors

**Health care workers’ attitude**

Two stated that quality of services rendered at ANC clinics depended on healthcare worker’s mood. They explained that ANC services should be made more attractive and more need centred. Current practice of just regular blood testing and issuing only vitamin and other supplements must change. The following are some responses:

‘You are not paying here. It’s free … if you are in a rush go for medical aid. They were so rude to treat a pregnant woman this way.’ (Participant 3, G1P0, 23 years)

‘Clinic nurse at my clinic turned me away … told me to come back, when I feel the baby moving…. I don’t like my clinic. Although it is far, I would like to come to this health centre because nurses do not shout here.’ (Participant 9, G1P0, 20 years)

Most women expressed this sentiment:

‘Nurses are good here but they are so busy … always helping but sometimes they shout when lots of women are in the clinic… This clinic needs more nurses.’

#### Long queues at antenatal care clinics

Overburdened health facilities with lack of health personnel are often a barrier to effective ANC services. The following responses attest to that:

‘It is so difficult for me to sit in a queue for a long time … especially when I am 7 month[*s*] pregnant. There is no appointment system in this clinic. I have to leave home early to fight the long queues.’ (Participant 8, G2P1, 22 years)

‘I spend a whole day for one visit due to this crowded place … because there is only one nurse to take care of all of us … we will be happy if they start an appointment system by telephone.’ (Participant 8, G2P1, 22 years)

The whole group acknowledged her proposal. Majority suggested that the problem of long queues could be solved by increasing staff numbers and starting an appointment system.

#### Communication to attend antenatal care clinics

All were asked the source of information about ANC services. Fourteen acknowledged that it was the mother or peers. None mentioned health care workers as the source of information, which was surprising. Eighteen women believed that ANC clinics are meant to check the baby, do HIV testing and to obtain the antenatal attendance card. There is a wide communication gap between health care workers and the community:

‘My mother told me that I should go to the clinic when she noticed that I was pregnant.’ (Participant 4, G1P0, 18 years)

‘I learnt about ANC when I heard from my friends that I should go to the clinic for a check-up.’ (Participant 18, G1P0, 29 years)

Focus group:

‘Nurse is only issuing ANC cards … they never told us what the right time is to come to the clinic… I didn’t know.’

Most women said that there should be individual or group information sessions for pregnant women.

### Patient-related factors

#### Unplanned and non-acceptance of pregnancy

Fourteen women said that the current pregnancy was unplanned. Pregnancy was a complete surprise. The observation is that an unexpected pregnancy leads to a late ANC booking:

‘This baby was a complete surprise, and I was not ready for it. I was so confused that when I came to my senses, it was already five months.’ (Participant 3, G1P0, 23 years)

‘I did have some bleeding and thought it was my periods as I tend to be irregular… It is only when I started feeling movements inside then I thought it might be a baby.’

‘I know I didn’t plan it. I have a 1 year old child … and now this one. I don’t know how to deal with it…. It’s so difficult.’ (Participant 18, G1P0, 29 years)

Most women agreed that it took some time to accept a pregnancy especially when you are not ready for a baby. Unplanned pregnancies often end with passive acceptance. They then become reluctant to visit ANC clinics.

When a woman says that she was not aware that she was pregnant, the reality lies between complete truth and absolute denial. It is influenced by emotional, social and economic circumstances. Four participants were shocked when they had an unplanned or an unexpected pregnancy. They were in denial until pregnancy was confirmed or becomes evident. This state led to late ANC booking:

‘I was shocked when pregnancy was confirmed. I didn’t want it but now I have accepted.’ (Participant 3, G1P0, 23 years)

#### Fear of parents’ reaction

Five out of the 20 participants claimed that they were scared to disclose their pregnancy to the parents. All of them were high school learners. They hid their pregnancies because they were scared that their sexual relationships would come out to the open:

‘I confirmed my pregnancy by testing at the chemist but didn’t do anything until my mother noticed that my abdomen was getting bigger. I was afraid to tell my mom because I am still schooling.’ (Participant 9, G1P0, 20 years)

Focus group:

‘There are many things we do not share with our parents … it’s not easy to disclose a pregnancy … you know, we never talk about these things with [*a*] parent.’

#### Avoiding HIV testing

Sixteen were aware that HIV testing is part of the prevention of mother-to-child transmission (PMTCT) programme of ANC services. Five participants booked ANC late because they were scared to test for HIV:

‘I knew about my pregnancy when I missed my first period. I was scared to test for HIV … you know there is no privacy when HIV testing at the clinic.’ (Participant 5, G4P3, 35 years)

‘I knew my HIV status but was afraid to disclose to my boyfriend … and you know if I came to the clinic they would test me and he would have got to know.’ (Participant 12, G1P0, 23 years)

Focus group:

‘The group discussion explored the community perception of late ANC bookings due to avoidance of HIV testing. Women accepted that testing for HIV does affect their relationship with boyfriends.’

### Socio-economic factors

#### Transport cost

Seventeen were unemployed and living under poor socio-economic conditions. They lived in remote areas with poor infrastructure:

‘I didn’t have money to come to the clinic. It is too far … and I can’t afford the transport cost of R250 for a private taxi.’ (Participant 13, G2P1, 28 years)

#### Partner neglect and loss of support

Eleven women claimed that their partners supported them financially. Unmarried women’s opinion was that father of the baby lost interest in them on disclosing their pregnancy. Therefore, many women did not want to disclose their pregnancy because they feared loss of support from the partner. They tend to hide their pregnancy until late and that led to late ANC booking:

‘I knew that my boyfriend didn’t really want this baby, so I hid it from him for a while…. I really just didn’t want to disclose it. If he discovered my pregnancy he will find another girl.’ (Participant 13, G2P1, 28 years)

Focus group:

‘You know, your boyfriend supports until you fell pregnant. Once they find out that you are pregnant, the next day they disappear. Now you have to go through it all alone’. Unmarried women expressed this sentiment.’

#### Working in another province

Two were working in other provinces and so had to delay ANC attendance. They attributed this to language barrier and also ignorance as to where the ANC facility was. Both desired to commence ANC services at a clinic closer to home:

‘I didn’t know much about Cape Town. I didn’t know where the clinic was…. I knew, I was going back home, so I decided to wait until I came here.’ (Participant 2, G2P1, 21 years)

### Individual perceptions

#### Experiences of previous pregnancy

Fourteen had one or more previous pregnancy experiences. Despite that none of the women knew about services rendered at an ANC clinic (PMTCT, urine tests, blood tests etc.). The responses reported on timing of ANC booking in the current pregnancy were directly related to outcome of the previous pregnancy. Women who had good outcomes during previous pregnancies had the following to say:

‘My mother told me to visit the clinic but I just ignored her because my last two children were born normally. In last pregnancy I booked at 8 weeks and this time I decided to come at 24 weeks … as I didn’t find any reason to visit early.’ (Participant 1, G3P2, 36 years)

‘Even last time I came at 20 weeks … no one told me that I was late.’ (Participant 17, G6P5, 38 years).

Twelve women perceive pregnancy as a normal physiological process and did not think there was any need to attend ANC early unless there was a medical condition:

‘During my first pregnancy I visited clinic early because I felt sick (Vomiting)…. This time there is no problem so I decided to come at six month*[s]*.’ (Participant 6, G3P2, 29 years)

Focus group:

‘I did not feel weird like during my first pregnancy…. I didn’t find any reason to attend ANC clinic’. People seek health care services mainly for medical reasons. Pregnant women had the same perception.’

‘It’s a common perception that if you delivered a baby without a problem then during next pregnancy you can relax … because it will be normal’.

Some participants argued that pregnancy is part of a women’s life (Focus group):

‘Since we knew labour process, early visit was unnecessary.’

#### Late booking had less antenatal care clinic visits

None was aware of the services offered to pregnant women at the ANC clinic. Three believed that if they book late, then they had to visit only a few times:

‘Nine months is too long, so there is no hurry to visit a clinic. I just needed the ANC card so that I can deliver at the hospital. Even at the last pregnancy I booked at 28 weeks and delivered a healthy baby.’ (Participant 2, G2P1, 21 years)

Focus group:

‘My friend told me, if I go early (first four month*[s]*) … I have to visit at least six-seven times. Especially multigravidae, who had healthy babies during the previous pregnancies, booked their current pregnancy late. They had a common perception that early ANC visit leads to too many subsequent visits.’

#### Other commitments

Five women did not consider antenatal visit to a clinic as a priority. They engaged in other work because pregnancy was perceived as a natural process for every adult woman:

‘I was busy with my studies and so didn’t have time to visit a clinic. Any how it is not necessary until my baby has grown big enough.’ (Participant 12, G1P0, 23 years)

### Failure of family planning

#### Using injectable contraception

A number of women claimed that they did not know that they were pregnant. Six of them had been on injectable contraception. Lack of knowledge, about risk of pregnancy, while using contraceptives delayed accessing ANC services:

‘I got my injection last year and it was a surprise to me when I visited the clinic for contraceptive injection and the nurse told me that I was pregnant.’ (Participant 8, G2P1, 22 years)

Focus group:

‘It is not easy to know whether one is pregnant or not … when you are on protection (Depo Provera) … because you are not having periods … sometimes bleeding is on and off.’

#### Difficulty in accessing termination of pregnancy

Five women did not want to continue with an unwanted pregnancy but failed to terminate because of difficulty in accessing services. Although the pregnancy is unwanted, they are forced to continue with the pregnancy which leads to delayed ANC booking. Most clinics do not offer medical termination of pregnancy. Two participants had resorted to backstreet abortions but the procedures had failed:

‘I didn’t want this baby. I went to the clinic and they said it’s too late. I even bought pills from the street but it did not work…. Now I am here because I didn’t have any choice.’ (Participant 14, G1P0, 19 years)

Focus group:

‘I came for an abortion…. They kept referring from one place to the other … I was tired and decided to keep the baby’. Women in the focus group suggested that each health centre should have facilities for termination of pregnancy.’

## Discussion

The decision of first ANC visit is more complex. It is influenced by how women perceive the best choices for themselves in their individual circumstances. This study identified four key themes to understand why women attend late ANC booking at Mbekweni area in South Africa. These include the factors of health care system, patient-related, socio-economic and individual perception and knowledge of ANC services.

### Health care system factors

Quality of care received at a clinic is reflected in pregnant women’s timing of an ANC visit. Poor and inadequate quality of care was a strong predictor for late ANC booking. Poor attitude, insensitivity and rudeness of health care workers often discouraged pregnant women attending ANC clinics. Insensitive responses from health care workers were also contributory to poor access of ANC services. Though most participants anonymously agreed that health care worker responses were better than what they expected, the communication between participant and health care worker during an ANC visit was limited and didactic. Similar results were also reported in other qualitative studies.^8,13,14^

Lack of coherent approach between the community and health care workers is a barrier to early ANC booking. The finding of this study is that women who had a perception of no benefit from early ANC attendance often postponed antenatal care. Therefore, community-based information, health education and communication on ANC services are important to overcome women’s perception of late booking. Lack of empowerment and passive acceptance of late visit for ANC is common amongst adolescent women.^15^ These results were in line with findings of studies by Daniels et al.^16^ and Tshabalala.^17^ Improving communication between the community and health care workers would promote the community to increase utilisation of ANC services.^2,18,19^

Women postponed their ANC visit because of unavailability and inaccessibility of health services. This study was conducted at Mbekweni Health Centre and it is one of the rural settings in Mthatha area. Rural areas are known to be poorly resourced and have fewer skilled health care workers. A study conducted by Isaac et al.^20^ also supported that woman in rural areas are more likely to book late because of unavailability of health services.^14^ Long waiting times and queues at ANC clinics were identified as contributing factors for late ANC visits. Women spending many hours sitting in queues after travelling long distances to see the health care workers discouraged them from attending ANC clinics.^20^

### Patient-related factors

Late recognition of pregnancy was one of the major reasons for late ANC bookings. This study further revealed that being unaware of pregnancy was a complex issue from lack of acceptance to non-recognition of early signs of pregnancy. There were two major responses. Women who did not haveamenorrhea and those who did not think that amenorrhea was caused by pregnancy. The resultant behaviours depended on the knowledge and previous experience of pregnancy. Young women often had difficulty in identifying, understanding and acknowledging physical signs of pregnancy. There was a small category of women who avoided pregnancy tests. These women had made no plan of care and refused to acknowledge the pregnancy. Similar findings were also reported by Daniels et al.^16^ and Johnson et al.^21^

Some were unaware of their pregnancy because they have been using long-acting injectable hormonal contraception. Women who used injectable contraceptives had hardly any knowledge of the risk of pregnancy while using contraception. Early recognition depended on personal knowledge and experience of pregnancy. Younger women with poor knowledge and awareness of pregnancy failed to recognise early signs of pregnancy.^8^ Women often misinterpret pregnancy signs when their perceived likelihood of falling pregnant is low. When some women have symptoms and signs of pregnancy, they tend to interpret them as being related to other medical conditions because they are on contraception. Women have an inability to recognise pregnancy symptoms while on contraception. They do not have a pregnancy mindset, so they do not expect any pregnancy symptoms.^19,22,23^

Unplanned pregnancies had a significant effect on late ANC bookings. Women delayed confirmation of pregnancy when they were not ready for another child. Ambivalences were the common reaction with unplanned pregnancies even when it was desired. Studies by Peacock et al.^24^ and Hulsey^25^ also support these findings. Similar findings also were reported in another South African study conducted by Sibeko and Moodley.^26^

Falling pregnant while using contraceptives shocked many women. This led them to accessing ANC late. Some delays were because they could not make up their mind to retain or to abort the foetus. Those who contemplated on termination of pregnancy initially, accepted to keep the pregnancy later, but reluctantly, because they failed to get to a place where the termination could be done safely. Better knowledge of reproductive health among women will promote early decision making in subsequent pregnancies.^19,27^

In 2010 SANDOH adopted the WHO recommendation of provider-initiated counselling and testing and client-initiated counselling and testing models as part of standard PMTCT programme. This replaced the existing voluntary counselling and testing model. Women often find out that they are living with HIV at the same time as their pregnancy is confirmed. They are often reluctant to share this information with partners and families. They fear discrimination, stigma, violence, abandonment and the other social consequences. Only a few studies have reported the stigma attached to HIV as a barrier to early ANC booking. We found that women were reluctant to come to ANC clinics to avoid HIV test. Because the HIV test is part of PMTCT, women postponed ANC visits to avoid HIV testing. Women also feared losing their partner if they disclosed their HIV status. Similar findings were reported in a Kenyan case report where women postponed ANC visit to avoid HIV testing.^13,18^

### Socio-economic factors

Women chose to seek care at a time convenient for them depending on their social circumstances, community perceptions and health care provider related issues. The social circumstances are financial dependence on others and easy accessibility of a health care facility. Financially insecure women were often unable to make informed decisions on their own. When women depend financially on their parents, partners or guardians, the decisions about pregnancy are often determined by them. This often leads to postponement of ANC bookings. Fear of the reaction of parents or guardians is also a determining factor for late ANC booking where women tend to hide their pregnancy.^17,28,29^

In this study it was found that if the partner and the family accept the pregnancy, then these women attend the ANC early. Fear of losing the partner if pregnancy was disclosed and being not sure whether to keep or abort the pregnancy lead to delayed ANC attendance. Other studies conducted also show that if the family and the society accept a pregnancy, then those women attend ANC much earlier.^16,21^ In some cases, the resulting anxiety led to denial of pregnancy. Timing of ANC visit was influenced by the willingness to accept the pregnancy.

The community perceptions are not recognising the usefulness of early bookings, multiparous women thinking that when earlier pregnancies have been normal, the subsequent one’s will be normal too and postponing of HIV testing until late in pregnancy because of personal fears.^13^

Some women who work in other provinces are in a dilemma whether to attend ANC where they work or in their home provinces. This is because of language barriers (e.g. isiXhosa speaker in the western province where Afrikaans is commonly spoken) and unfamiliarity of the place. Women preferred to attend ANC at their native place and so they stay away from the clinic when they work away from home. They had feelings of insecurity and lack of understanding of the strange place when they worked away from home. They preferred to postpone ANC visits until they returned to their place of safety and trust. Thus, working away from home is a barrier to early access of ANC. Study by Rosalind et al.^21^ also reported, being ‘on the move’ as a barrier for early ANC booking.^20,30^

### Individual perception and knowledge

Experiences of past pregnancies had a direct impact on the timing of ANC bookings. Women who had positive outcomes postponed their ANC visit because they did not perceive any benefit from early ANC booking. Women who attended ANC in the first trimester in the previous pregnancy did not do it again. As a result of lack of empowerment, women did not perceive the pregnancy as requiring immediate medical attention. These findings were supported by other studies conducted by Haddrill et al.^21^ and Tariku et al.^31^

Many study participants did not perceive any benefit from early ANC bookings. They believed that this booking is only to receive the attendance card. Collecting the card was more important than timing of the visit. Women had common perceptions that early booking led to more visits. They preferred less visits by postponing ANC attendance. Similar findings were also reported in other South African studies by Jewkes et al.^32^ and Myer and Harrison^33^ where women did not perceive benefits of early booking and risks associated with late bookings. Some women had other commitments in life. Women identified antenatal services as important part of pregnancy but not as an immediate one. When women had other commitments, they decided to postpone and delay ANC booking.^16,17,34^ Women will attend ANC early once they start to perceive benefits of early bookings. Knowledge empowerment can mobilise women to seek early ANC visits.^32,33^

Most of the women perceived pregnancy as a natural physiological process and postponed ANC visits until any medical condition forced them to visit a health facility. Many studies have reported that women did not feel the need for ANC visits when they did not encounter any problems during pregnancy. Similar results have been reported by Jewkes et al.^32^ and Okunlola et al.^35^ According to Myer and Harrison,^33^ women did not perceive pregnancy as an immediate health risk and in turn their view was that early booking was not necessary.^32,33,34,35^ In this study most women did not know the benefits of early ANC booking.

### Strength of this study

The qualitative design included in-depth interviews and focus group discussions to understand women’s perception of late ANC bookings. Focus group discussions helped in uncovering the multidimensional views behind timing of ANC visits.

### Limitation of this study

This study was conducted at a health centre and therefore there is a possibility of underreporting of health care worker related factors for delayed ANC booking. The study also considered reflexivity and researcher bias during focus group interviews. The researcher was present during focus group discussion. He is a family physician and provides support for ANC services at the Mbekweni Health Centre. Though the focus group discussion was led by research assistance, participant’s response towards health care worker related factors might be influenced by the presence of the researcher. The study reported the views of those who attended the ANC clinic. There are women who never attend ANC clinic and their views may be different. The study did not include teenage pregnancies who are likely to book late for their ANC visits.

## Conclusion

The timing of first ANC booking was influenced by a spectrum of causes from lack of health system support to pregnant women’s personal beliefs, knowledge and perceptions regarding antenatal services. Shortage of health care personal and lack of active engagement with pregnant women encourage late ANC visit. Unwanted pregnancies were common as a result of poor access to contraceptive services and as a result of lack of choice of termination of pregnancy; most of them were attend with late ANC booking. Lack of reproductive health knowledge in primigravidae and previous experience of low-risk previous pregnancy in multigravidae promotes late ANC visit. Financial independency had direct influence on disclosure and subsequent timing of ANC booking. Accessibility and affordability of ANC services utilisation in rural areas is still a common problem.
